# CX3CR1-Expressing Immune Cells Infiltrate the Tumor Microenvironment and Promote Radiation Resistance in a Mouse Model of Lung Cancer

**DOI:** 10.3390/cancers15225472

**Published:** 2023-11-19

**Authors:** Tamar Ben-Mordechai, Yaacov R. Lawrence, Zvi Symon, Ariel Shimoni-Sebag, Uri Amit

**Affiliations:** 1 Radiation Oncology Department, Chaim Sheba Medical Center, Ramat Gan 52621, Israel; tammybm24@gmail.com (T.B.-M.); yaacov.lawrence@sheba.health.gov.il (Y.R.L.); zvi.symon@sheba.health.gov.il (Z.S.); ariel.sebag@gmail.com (A.S.-S.); 2 Faculty of Medicine, Tel Aviv University, Tel Aviv 69978, Israel; 3 Radiation Oncology Department, Tel Aviv Medical Center, Tel Aviv 64239, Israel; 4 Department of Radiation Oncology, Perelman School of Medicine, University of Pennsylvania, 3400 Civic Center Boulevard, TRC 2 West Philadelphia, Philadelphia, PA 19104, USA

**Keywords:** radiation, immunotherapy, cytokines, lung cancer

## Abstract

**Simple Summary:**

CX3CR1 is present in a subset of the immune cells in the tumor microenvironment; however, their function after radiation therapy remains unknown. We found that radiation induces a significant influx of CX3CR1-expressing immune cells, notably macrophages, into the tumor microenvironment. Co-culturing irradiated lung carcinoma cells with CX3CR1-deficient macrophages reduced proliferation and increased apoptosis of the cancer cells. Interestingly, deficiency of CX3CR1 in macrophages led to the redistribution of the irradiated cancer cells in the S-phase, parallel to increased expression of cyclin E1, required for cell cycle G1/S transition. In addition, deficiency in CX3CR1 in macrophages altered cytokine secretion with decreased interleukin 6, a mediator of cancer cell survival and proliferation. Lastly, we show that in vivo ablation of CX3CR1-expressing cells attenuates tumor progression following radiation and sensitizes the tumor to S-phase-specific chemotherapy.

**Abstract:**

Introduction: Chemokine (C-X3-C Motif) Receptor 1 (CX3CR1) is present in a subset of the immune cells in the tumor microenvironment (TME) and plays an essential and diverse role in cancer progression. However, its potential function in the irradiated TME remains unknown. Materials and Methods: A mouse lung cancer model was performed by subcutaneously inoculating Lewis Lung Carcinoma (LLC) cells expressing luciferase (Luc-2) and mCherry cells in CX3CR1^GFP/GFP^, CX3CR1^DTR/+^, and wild–type (WT) mice. Bioluminescence imaging, clonogenic assay, and flow cytometry were used to assess tumor progression, proliferation, and cell composition after radiation. Results: Radiation provoked a significant influx of CX3CR1-expressing immune cells, notably monocytes and macrophages, into the TME. Co-culturing irradiated LLC cells with CX3CR1-deficient monocytes, and macrophages resulted in reduced clonogenic survival and increased apoptosis of the cancer cells. Interestingly, deficiency of CX3CR1 in macrophages led to a redistribution of the irradiated LLC cells in the S-phase, parallel to increased expression of cyclin E1, required for cell cycle G1/S transition. In addition, the deficiency of CX3CR1 expression in macrophages altered the cytokine secretion with a decrease in interleukin 6, a crucial mediator of cancer cell survival and proliferation. Next, LLC cells were injected subcutaneously into CX3CR1^DTR/+^ mice, sensitive to diphtheria toxin (DT), and WT mice. After injection, tumors were irradiated with 8 Gy, and mice were treated with DT, leading to conditional ablation of CX3CR1-expressing cells. After three weeks, CX3CR1-depleted mice displayed reduced tumor progression. Furthermore, combining the S-phase-specific chemotherapeutic gemcitabine with CX3CR1 cell ablation resulted in additional attenuation of tumor progression. Conclusions: CX3CR1-expressing mononuclear cells invade the TME after radiation therapy in a mouse lung cancer model. CX3CR1 cell depletion attenuates tumor progression following radiation and sensitizes the tumor to S–phase-specific chemotherapy. Thus, we propose a novel strategy to improve radiation sensitivity by targeting the CX3CR1-expressing immune cells.

## 1. Introduction

Radiotherapy is one of the cornerstone treatment modalities for cancer, with more than 60% of patients receiving radiation treatment [1]. Traditionally, it was thought that radiation kills tumor cells by causing double-strand DNA breaks. However, recent evidence suggests that the cytolytic effect is also mediated by modifying the tumor microenvironment (TME) and facilitating immunogenic cell death [2,3]. This potential synergy is the rationale for current clinical trials combining radiation with immune checkpoint inhibitors [1,4]. However, ionizing radiation can also elicit the attraction of immunosuppressive cells and the release of anti-inflammatory cytokines [5], contributing to radiation resistance. Thus, improving the efficacy of radiation therapy requires a better understanding of the immunomodulatory roles of the immune cells within the irradiated TME [6,7].

Chemokine (C-X3-C Motif) Receptor 1 (CX3CR1) is a member of the seven-transmembrane G-protein coupled receptors present on a subset of immune cells. It is a receptor for its sole ligand, CX3C ligand 1 (CX3CL1), called fractalkine (FKN) [8], and mediates the chemotaxis of immune cells, most notably monocytes and macrophages but also dendritic cells, lymphocytes, and natural killer cells [9]. CX3CR1 signaling has been studied in cancer and plays conflicting pro and antitumor functions. CX3CL1 expression correlates with prolonged survival in lung adenocarcinoma patients [10], and in a mouse model of melanoma, CX3CR1-deficient mice displayed larger tumors relative to their wild-type (WT) counterparts [11]. In contrast, other studies showed that the CX3CR1 signaling could promote cancer proliferation, cell cycle progression, and resistance to apoptosis [12,13,14]. Despite evidence that CX3CR1-expressing immune cells in the TME play an essential and diverse role in cancer progression, its potential role in radiation response remains unknown.

## 2. Materials and Methods

All experimental protocols were approved by the Animal Care and Use Committee of Chaim Sheba Medical Center, Tel-Aviv University (Israeli Ministry of Health approval number 11843). All methods are reported in accordance with ARRIVE guidelines (https://arriveguidelines.org) (accessed on 1 January 2021) for the reporting of animal experiments.

### 2.1. Cell Line and Animals

The LLC cell line was kindly received from Dr. Pablo Blinder’s lab at Tel-Aviv University, Tel-Aviv, Israel. These LLC cells express luciferase (Luc-2) and mCherry. To investigate the role of CX3CR1 expressing immune cells, we used CX3CR1^GFP/GFP^ [15]. In these genetically engineered mice, the murine *CX3CR1* gene was replaced with the gene encoding the enhanced green fluorescent protein. This approach allows the generation of mice lacking CX3CR1 while enabling the study of CX3CR1 expression patterns and migration of cells that express this receptor [15,16,17,18,19]. To determine the CX3CR1-deficient immune cells’ effect on irradiated LLC in vivo, we used CX3CR1^DTR/+^ mice. These mice express the diphtheria toxin receptor (DTR) under the *CX3CR1* promoter. Given diphtheria toxin (DT), CX3CR1-expressing cells expressing DTR are selectively ablated by apoptosis due to the DTR-DT bond [17]. Prof. Steffen Jung’s lab kindly provided both CX3CR1^GFP/GFP^ and CX3CR1^DTR/+^ C57BL/6J transgenic mice. C57BL/6J WT mice were obtained from Harlan Laboratories (Jerusalem, Israel). CX3CR1-deficient monocytes and macrophages were harvested from CX3CR1GFP/GFP mouse peritoneum following injection of thioglycolate, intraperitoneal (IP) to 12-week-old CX3CR1^GFP/GFP^ mice (20 g.). Three days later, mice were anesthetized with 2% isoflurane, and sterile PBS (2 mL) was injected into their abdominal cavity to aspirate the peritoneal fluid. Following centrifugation, the pellet of peritoneal macrophages was subjected to further experiments [20].

### 2.2. Radiation Source and Procedure

Cells and mice were irradiated using the IC-320 biological irradiator (Kimtron, Oxford, CT, USA) at 270 kV, 7 mA at an SSD 70 cm at a dose rate of 0.46 Gy/min. Mice were anesthetized with a combination of ketamine (80 mg/kg) and xylazine (10 mg/kg) IP to provide a 20–30 min sedation. Each mouse was placed in a confined lead casing, in a ventral recumbent position, with its tumor-bearing hind limb centered in the middle of a 1 cm round hole in the lead casing to allow for localized tumor irradiation. Following radiation, mice were placed on a heating pad and allowed full recovery before being returned to their cages.

### 2.3. Tumor Formation, Detection, and Monitoring

For tumor formation, 750,000 LLC cells were inoculated subcutaneous (SC) in mouse hind limbs. We irradiated tumors with 8 Gy at a size of 5 mm × 5 mm, which was measured using a caliper. For in vivo tumor growth monitoring, we injected D-luciferin (150 mg/1 kg; Regis Technologies Inc., Morton Grove, IL, USA), IP 10 min before imaging the mice using an In vivo Imaging System (IVIS; Perkin Elmer, Ra’anana, Israel). Mice were anesthetized by inhalation of 2% isoflurane. The bioluminescence of tumors was detected and analyzed by Living Image in vivo 4.5.4 software (Perkin Elmer, Ra’anana, Israel).

### 2.4. Characterization of Tumor Immune Cells

We used flow cytometry to determine the immune cell profile (Cytoflex, Beckman Coulter, Indianapolis, IN, USA). We harvested the tumors and put them through 20 min of digestion by an enzyme cocktail [21]. The purified cells were centrifuged and processed for flow cytometry. Cells were stained with PE-conjugated anti-mouse CX3CR1 (BioLegend, San Diego, CA, USA) and APC/Cy7 conjugated anti-mouse CD45 (BioLegend, San Diego, CA, USA) for desired population definition. To further characterize immune cell composition in the TME, the cells were also stained with PE/Cy7 conjugated anti-mouse Ly6G, PerCP conjugated anti-mouse Ly6C, FITC conjugated anti-mouse F4/80, APC conjugated anti-mouse CD11b, APC conjugated anti-mouse CD335, APC conjugated anti-mouse CD3, and PerCP/Cy5.5 conjugated anti-mouse CD19 (BioLegend, San Diego, CA, USA). Analysis was performed using CytExpert 2.3 software.

### 2.5. Clonogenic Assay

To determine the CX3CR1-deficient macrophages’ effect on irradiated LLC cells, we isolated peritoneal macrophages from WT and CX3CR1^GFP/GFP^ mice. Briefly, mice were anesthetized, and the peritoneal cavity was exposed and washed with saline. Peritoneal fluid was centrifuged for 8 min at 1600 rpm at 4 °C. Cells went through the Easysep (Enco, Petach Tikvah, Israel) kit with F4/80 or CX3XR1 antibody for macrophage isolation. We then cultured 125,000 peritoneal macrophages per well in a six well culture dish. We irradiated LLC cells with 6 Gy and transferred 1000 cells to each well and co-cultured with peritoneal macrophages. To further enhance CX3CR1-expressing cell activity, we added FKN 100 μg/mL (BioLegend, San Diego, CA, USA). Following eight days of co-culture, cells were stained with crystal violet (Sigma-Aldrich, Rehovot, Israel) for colony detection. Colonies, defined as containing at least 50 cells, were counted under a microscope.

### 2.6. Cell-Cycle and Apoptosis

We analyzed cell-cycle and apoptosis rates to characterize irradiated LLC following co-culture with CX3CR1-deficient macrophages. Cell-cycle was determined following cell staining with propidium iodide (PI) and analysis by Cytoflex flow cytometry. Apoptosis was determined by co-staining the cells with annexin-PI using the MEBCYTO apoptosis kit (MBL, Woods Hole, MA, USA).

### 2.7. Real-Time PCR

Total RNA was purified from irradiated LLC using Direct-zol™ RNA MiniPrep (Zymo Research, CA, USA) according to the manufacturer’s protocol. Total LLC RNA (200 μg) was reverse transcribed into cDNA using the M-MLV reverse transcriptase (Promega, Madison, WI, USA). The expression of cyclin-dependent kinase 1 (CDK1), CDK2, CDK4, cyclin A2, cyclin B1, cyclin D1, and cyclin E1 was determined with SYBR Green PCR Master Mix (Life Technologies, Carlsbad, CA, USA). Gene expression was normalized to the housekeeping gene glyceraldehyde 3-phosphate dehydrogenase (GAPDH). Data were analyzed using the DDCt method with the aid of the StepOnePlus Software v2.2.2 (AppliedBiosystems, Foster City, CA, USA). Primers are listed in Supplementary Table S1.

### 2.8. Cytokines Assays

To determine the secretory profile of CX3CR1-deficient and WT macrophages, we subjected cell supernatant to Quansys Q-plex Array Chemiluminescent (Quansys Biosciences Multiplex ELISA, Quansys Biosciences, West Logan, UT, USA), to detect the level of 16 different mouse cytokines. Conditioned immune cells were incubated for 1 h in a 96-well plate printed with 16 antibodies in each well. Then, a detection mix was added for 1 h, followed by streptavidin-HRP incubation for 15 min, and a substrate solution addition was followed by immediate imaging using the Quasys Q-ViewTM system and Q-View version 3 software.

To measure CX3CL1 chemokine levels in conditioned medium from CX3CR1-deficient and WT macrophages, the Mouse Fractlakine ELISA Kit (ThermoFisher, EMCX3CL1 Waltham, MA, USA) was used. The conditioned medium was incubated for 2.5 h in a 96-well plate coated with the capture antibody in each well. Then, a detection mix was added for 1 h, followed by streptavidin-HRP incubation for 45 min and a substrate solution addition. The absorbance was measured at the prespecified wavelength of 450 nm, according to the manufacturer’s instructions.

### 2.9. Experimental CX3CR1^DTR/+^ LLC Mouse Model

We inoculated 750,000 LLC cells into CX3CR1^DTR/+^ or WT mice hindlimbs. After the tumor reached a size of 5 mm × 5 mm, we irradiated all mouse tumors with a single fraction of 8 Gy and divided them into four groups: 1. WT mice; 2. CX3CR1^DTR/+^ mice; 3. Gemcitabine injection in WT mice; 4. Gemcitabine injection in CX3CR1^DTR/+^ mice. The S-phase specific chemotherapeutic gemcitabine (30 mg/kg body weight) was injected IP, 1- and 8-days following radiation. DT (4 μg/g body weight) was injected IP into all mice every three days. Mouse tumors were imaged once a week by IVIS 10 min after IP injection of D-luciferin (150 mg/1 kg). 

### 2.10. Statistical Analysis

Statistical analysis was performed using GraphPad Prism version 10.00 for Windows (GraphPad Software, San Diego, CA, USA) for all the cell and mouse experiments. Differences between the two groups were compared by the Student *t*-test or the Mann–Whitney test, where data were not normally distributed. One-way ANOVA with Bonferroni correction was used to compare differences in cytokine secretion, gene expression and clonogenic assays. Two-way repeated-measures ANOVA with Bonferroni correction was used to test whether measurements of total flux over the tumor area varied over time among the experimental groups. Each experiment was performed once. Differences were considered significant at a *p* < 0.05.

## 3. Results

### 3.1. CX3CR1-Expressing Immune Cells Infiltrate the TME following Radiation

**Figure 1:**
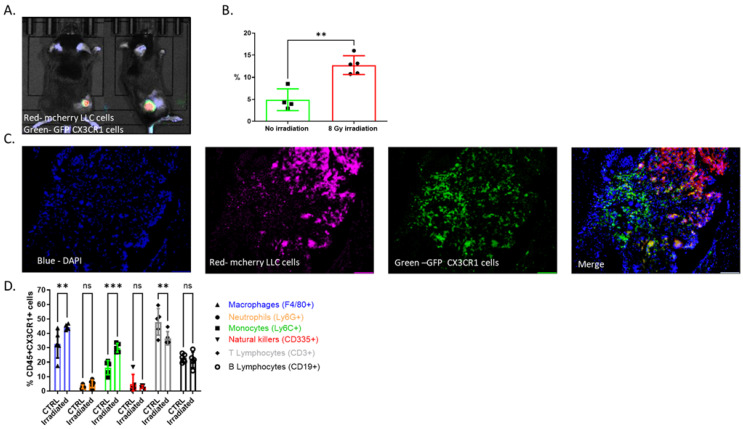
CX3CR1-expressing immune cells infiltrate the TME after radiation. (**A**) LLC cells (750,000) were injected SC into mice. Three weeks following injection, tumors were irradiated at 8 Gy. One week after irradiation, tumors were imaged using IVIS. (**A**) representative bioluminescence image of an irradiated mouse. LLC cells are shown in red (mCherry), and CX3CR1 immune cells within the tumor are green (GFP). (**B**) The percentage of CX3CR1-expressing cells within mouse tumors was significantly increased following irradiation with 8 Gy. Cells were imaged by flow cytometry using their GFP expression. Tumors without irradiation (no irradiation), *n* = 4; 8 Gy irradiated tumors (8 Gy irradiation), *n* = 5. (**C**) Fluorescent microscopy image showing infiltration of CX3CR1 expressing cells (GFP) in the irradiated TME (mCherry). (**D**) Cells from irradiated (8 Gy; irradiated) or not (CTRL) LLC tumors were isolated three days after irradiation, stained for immune cell markers, and subjected to flow cytometry. The markers for immune cell characterization were as follows: CD45 and CX3CR1 for population definition; Ly6G for neutrophils (N; orange); Ly6C for monocytes (MN; green); F4/80 and CD11b for macrophages (MQ; blue); CD335 for natural killer cells (NK; red); CD3 for T lymphocytes (T; gray) and CD19 for B lymphocytes (B; black; ** *p* < 0.01; *** *p* < 0.001; ns—not significant.

We began by characterizing CX3CR1 expression in the TME following radiation. A mouse lung cancer model was created by subcutaneously inoculating Lewis Lung Carcinoma (LLC) cells expressing luciferase (Luc-2) and mCherry in female CX3CR1^GFP/GFP^ reporter mouse hind limbs. After two weeks, mice were randomized to a treatment group in which visible tumors received a single fraction of 8 GY and a control group that did not receive radiation. As expected, tumor growth was attenuated in the irradiated mice). After irradiation, CX3CR1-expressing immune cells accumulated at the TME (Figure 1A). This increase in CX3CR1-expressing immune cells was significantly higher than in tumors that were not irradiated (Figure 1B, C). To further characterize infiltrating CX3CR1-expressing immune cells, we used flow cytometry (FACS) for an array of cell markers and compared cell profiles between irradiated and non-irradiated tumors (Figure 1D). Among the CX3CR1-expressing immune cells (CD45^+^), we could detect monocytes, macrophages, T and B cells at a high rate, while neutrophils and natural-killers (NK) showed low expression of CX3CR1 (Figure 1D). Following radiation, we detected an elevation in CX3CR1-expressing monocytes and macrophages and a decrease in CX3CR1-expressing T cells. The cell percentage of CX3CR1-expressing neutrophils, NK, and B cells was not altered following radiation (Figure 1D).

### 3.2. Deficiency of CX3CR1 in Macrophages Enhances LLC Cells’ Radiation Sensitivity In Vitro

**Figure 2:**
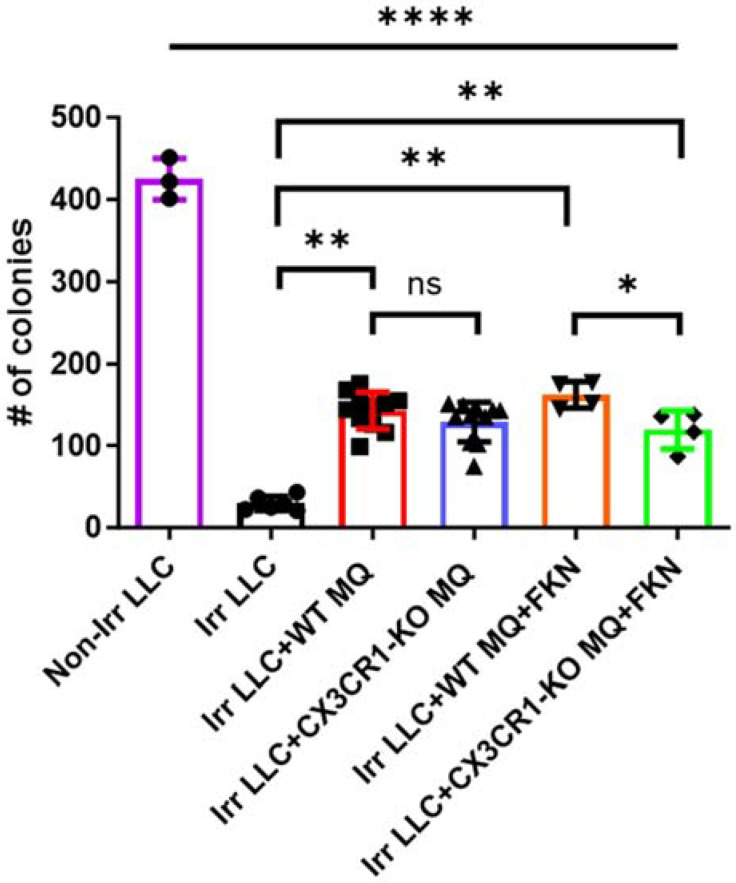
Deficiency of CX3CR1 in macrophages enhances LLC cells’ radiation sensitivity in vitro. The number of LLC colonies was significantly lower after irradiation. The number of irradiated LLC cell colonies increased after co-culture with WT macrophages and FKN compared to co-culture with CX3CR1-deficient macrophages and FKN. * *p* < 0.05; ** *p* < 0.01; **** *p* < 0.0001. FKN—fractalkine; Irr—irradiated; MQ—macrophages; ns—not significant; WT—wild-type.

To determine the role of CX3CR1 in the TME following irradiation, we isolated peritoneal monocytes and macrophages from CX3CR1-deficient (CX3CR1^GFP/GFP^) or WT mice and co-cultured them with irradiated LLC cells. The FACS analysis showed that 60% of the WT macrophages express CX3CR1 (Supplementary Figure S1A,B). We tested the CX3CR1-expressing macrophages’ effect on irradiated LLC cells using the clonogenic assay. Co-culture with macrophages (CX3CR1-deficient or WT) increased the number of LLC colonies compared with the control of only irradiated LLC cells (Figure 2). The addition of FKN, the only ligand of CX3CR1, to WT macrophages and co-culturing with irradiated LLC increased the cancer colony number compared to co-culture of irradiated LLC with CX3CR1-deficient macrophages treated with FKN (Figure 2).

### 3.3. Co-Culture of CX3CR1-Deficient Macrophages with Irradiated LLC Cells Alters Cell Cycle Distribution, Regulation, and Apoptosis

**Figure 3:**
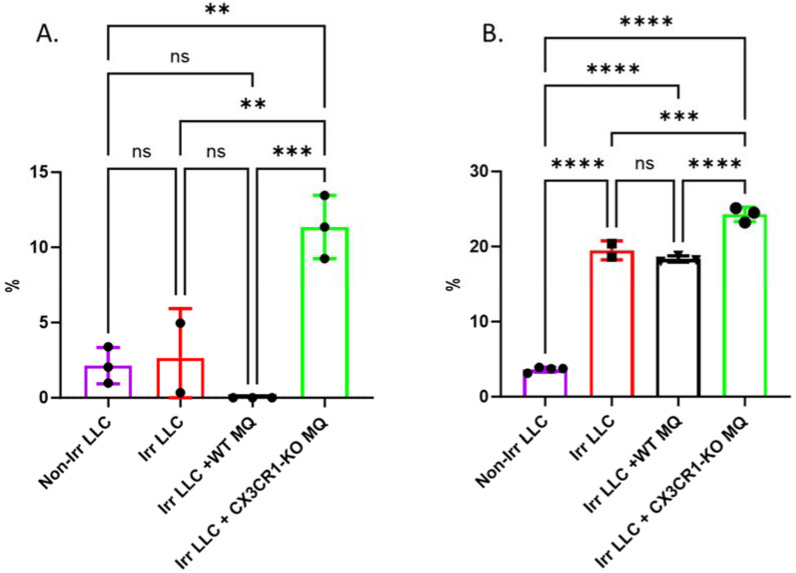
Irradiated LLC cells co-cultured with CX3CR1-deficient macrophages show increased cell death due to apoptosis. Apoptosis was determined three days after irradiated LLC cells were co-cultured with WT macrophages or CX3CR1-deficient macrophages: (**A**) Early apoptosis was determined by annexin positive and PI negative staining. (**B**) Late apoptosis was determined by annexin and PI co-staining. ** *p* < 0.01; *** *p* < 0.001; **** *p* < 0.0001; ns—not significant.

Radiation of cancer cells induces DNA damage, activating checkpoint pathways which, in turn, impede the movement of cells through the G1- and G2-phases and causing a delay in passage through the S-phase [22,23]. Checkpoints allow for DNA damage repair or may cause the cell to undergo apoptosis if the damage cannot be repaired [24]. Therefore, we tested the cell cycle of irradiated LLC cells and cell death following co-culture with CX3CR1-deficient macrophages. Cells were stained with annexin and PI to determine whether co-culture with CX3CR1-deficient macrophages affects LLC cells’ death following radiation. Accordingly, apoptosis was significantly higher among irradiated LLC co-cultured with CX3CR1-deficient macrophages than irradiated LLC co-cultured with WT macrophages or cultured irradiated LLC cells only (Figure 3A,B).

**Figure 4:**
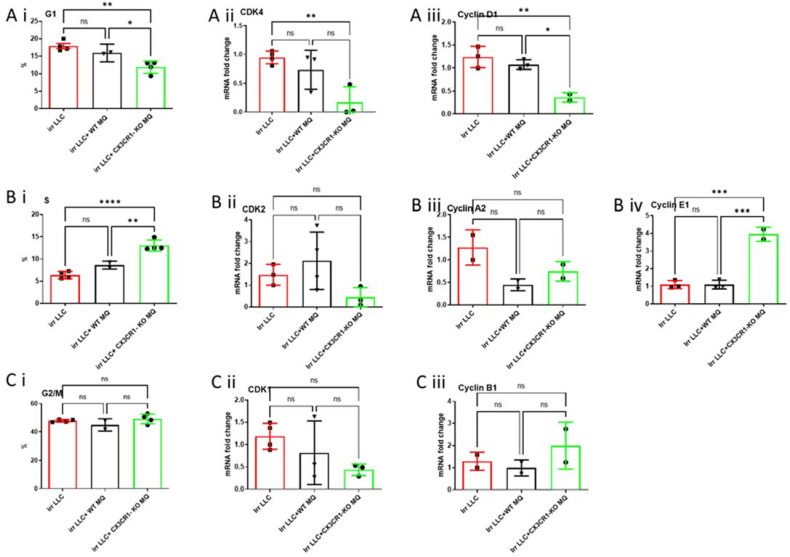
Co-culture of irradiated LLC cells with CX3CR1-deficient macrophages results in altered cell cycle distribution and regulation compared to co-culture with WT macrophages. Irradiated LLC cells co-cultured with CX3CR1-deficient macrophages showed a decrease in cell rate at G1-phase (**Ai**), no difference in CDK 4 expression (**Aii**), and low cyclin D1 expression (**Aiii**). Irradiated LLC cells co-cultured with CX3CR1-deficient macrophages showed an increase in cell rate at S-phase (**Bi**), no difference in CDK 2 expression (**Bii**), no difference in cyclin A2 expression (**Biii**), and high cyclin E1 expression (**Biv**). Irradiated LLC cells co-cultured with CX3CR1-deficient macrophages showed no difference in cell rate at G2–M-phases (**Ci**), no difference in CDK1 expression (**Cii**), and no difference in Cyclin B1 expression (**Ciii**). All reactions were run as triplicates, and each dot represents the mean value. * *p* < 0.05; ** *p* < 0.01; *** *p* < 0.001; **** *p* < 0.0001; ns—not significant.

The percentage of irradiated LLC cells found in the G1-phase was the lowest following co-culture with CX3CR1-deficient macrophages (Figure 4(Ai)). Moreover, expression levels of CDK4 and cyclin D1, which regulate the G1-phase, were also the lowest in these cells (Figure 4(Aii,Aiii)). We detected an increased irradiated LLC percentage in the S-phase after co-culture with WT immune cells; nevertheless, it was lower than LLC cultured with CX3CR1-deficient macrophages (Figure 4(Bi)). The increase in LLC cells in the S-phase in plates cultured with CX3CR1-deficient macrophages was parallel to increased expression of cyclin E1, whose activity is required for cell cycle G1/S transition (Figure 4(Bi–Biv)). Co-culture of CX3CR1-deficient macrophages with irradiated LLC cells did not alter the percentage of LLC cells in the G2–M-phase, compared with culture of irradiated LLC with WT macrophages (Figure 4(Ci)). Accordingly, cell cycle G2–M-checkpoint regulatory genes CDK1 and cyclin B1 expression levels did not differ among the three groups (Figure 4(Cii,Ciii)).

### 3.4. Deficiency in CX3CR1 in Macrophages Results in Increased Proinflammatory Cytokine Secretion

**Figure 5:**
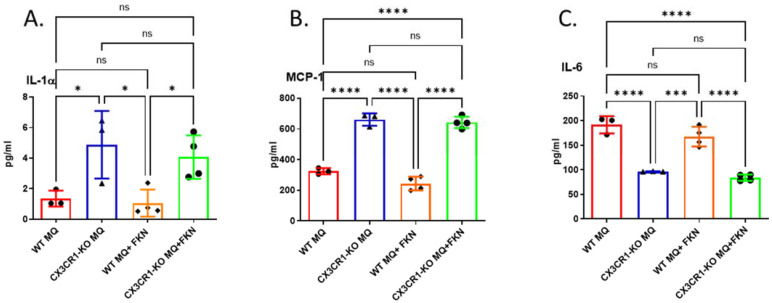
Deficiency of CX3CR1 in macrophages results in increased proinflammatory cytokine secretion. Conditioned medium of WT or CX3CR1-deficient macrophages was subjected to cytokine multiplex analysis for cell characterization. CX3CR1-deficient macrophages showed a significant increase in proinflammatory cytokines IL-1α (**A**) and MCP-1 (**B**) and a significant decrease in IL-6 (**C**) secretion, compared with WT macrophages with or without the addition of FKN. * *p* < 0.05; *** *p* < 0.001; **** *p* < 0.0001; ns—not significant.

Next, we sought to determine the CX3CR1-deficient macrophages’ cytokine secretion profile. We analyzed the conditioned medium of WT or CX3CR1-deficient macrophages 24 h after culturing by a multiplex array of 16 cytokines. Three key cytokines showed altered expression levels: interleukin (IL)-1α, MCP-1, and IL-6. CX3CR1-deficient macrophages showed a significant increase in proinflammatory cytokines IL-1α and MCP-1 with or without the addition of FKN (Figure 5A, B), compared with WT macrophages. However, IL-6, a crucial mediator of cancer progression, proliferation, and chemoresistance [25], was significantly decreased in CX3CR1-deficient macrophages compared with WT macrophages (Figure 5C). There were no significant differences in tumor necrosis factor α (TNF-α) and CX3CL1 secretion between WT and CX3CR1-deficient macrophages (Figure S3).

### 3.5. Conditional Ablation of CX3CR1-Expressing Immune Cells and S-Phase Specific Chemotherapeutic Gemcitabine in the Irradiated TME Significantly Attenuates LLC Tumor Progression In Vivo

**Figure 6:**
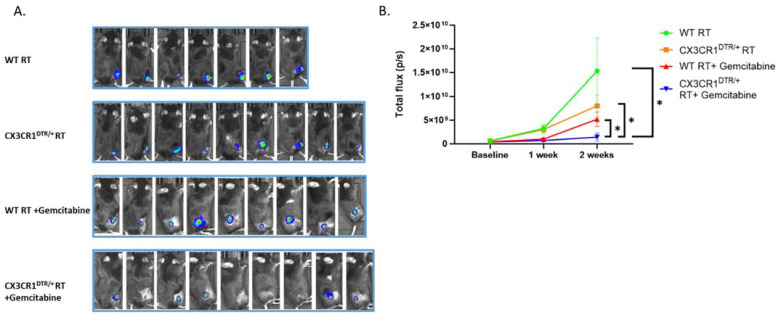
Ablation of CX3CR1-expressing immune cells after radiation attenuates tumor growth and sensitizes cancer cells to the S-phase-specific chemotherapeutic gemcitabine. (**A**) LLC cells expressing luciferase were injected subcutaneously into CX3CR1^DTR/+^ mice, sensitive to diphtheria toxin (DT), and WT mice. After a week, all the tumors were irradiated (RT) with 8 Gy, followed by IP injection of DT in all mice every three days, and division into four groups: WT mice, CX3CR1^DTR/+^ mice, WT mice treated with gemcitabine, and CX3CR1^DTR/+^ mice treated gemcitabine. Tumor progression kinetics were assessed using IVIS. (**B**) Ablation of CX3CR1 expressing immune cells decreased tumor growth (orange line; n = 9) compared with WT mice (green line; *n* = 7). Injection of gemcitabine in addition to CX3CR1 expressing cell ablation after irradiation further attenuated tumor growth (blue line; *n* = 9). * *p* < 0.05.

To determine the role of CX3CR1 in vivo and its ablation effect on tumor progression, we used CX3CR1^DTR/+^ mice sensitive to diphtheria toxin (DT). In this mouse model, when given DT by IPinjection, the cells go through apoptosis, and CX3CR1- expressing cells are ablated. First, we subcutaneously injected 750,000 LLC cells into CX3CR1^DTR/+^ and WT mice for tumor formation. After a week, the tumors were irradiated with 8 Gy, followed by IP injection of DT to all mice every three days. Analyzing immune cells from the tumors after irradiation and DT injections revealed a decrease in CX3CR1-expressing immune cells by flow cytometry (Supplementary Figure S2A). FACS analysis of the LLC tumors did not show a difference in the percentage of macrophages (CD45^+^ F4/80^+^) in the irradiated TME after CX3CR1 cell ablation (Supplementary Figure S2B). However, further characterization into M2-protumorigenic (also known as tumor-associated macrophages, TAM) and M1-antitumorigenic phenotypes showed a significant reduction in the M2 (CD45^+^ F4/80^+^ CD206^+^) with no difference in the percentage of M1 (CD45^+^ F4/80^+^ CD86^+^). Next, we aimed to determine whether ablation of CX3CR1 expressing immune cells reduces tumor progression and whether the redistribution of cancer cells in the S-phase can be exploited by adding the S-phase specific chemotherapeutic gemcitabine. We injected LLC cells to generate tumors in WT or CX3CR1^DTR/+^ mouse hind-limbs. One week later, mice tumors were irradiated (8 Gy) and divided into four treatment groups: WT mice, CX3CR1^DTR/+^ mice, WT mice treated with the gemcitabine, and CX3CR1^DTR/+^ mice treated with gemcitabine (Figure 6A,B). We followed the mice for two additional weeks until tumors in the WT group exceeded the authorized volume. While WT mice showed increased tumor progression, transgenic mice with conditional CX3CR1 cell ablation displayed attenuated tumor progression. Treating WT mouse tumors with gemcitabine decreased tumor growth (Figure 6B). However, the most profound effect was demonstrated in transgenic mice with CX3CR1 expressing cells, ablation treated with gemcitabine (Figure 6B). In this group, three mice had a complete tumor regression at the end of the follow-up (Supplementary Figure S4D).

## 4. Discussion

Combining radiation and immunotherapy targeting T lymphocytes is being actively studied in lung cancer treatment. However, the role of other immune cells in the irradiated TME remains largely unknown. Our study provides new evidence that CX3CR1-expressing immune cells play a significant role in radiation resistance in a murine lung cancer model. We demonstrated that CX3CR1-expressing immune cells, especially monocytes and macrophages, invade the TME after radiation. Deleting CX3CR1 expression in macrophages alters the secretion profile with a higher secretion of inflammatory cytokines IL-1α and MCP-1 and a significant decrease in IL-6, a crucial mediator of cancer cell survival and proliferation. In vitro, the co-culture of irradiated LLC with CX3CR1 deficient macrophages reduces cancer cells’ proliferation and induces cell-cycle dysregulation and apoptosis.

Interestingly, co-culture with CX3CR1-deficient macrophages led to the redistribution of the irradiated LLC cells in the S-phase. Furthermore, combining the S-phase specific chemotherapeutic gemcitabine with the conditional ablation of CX3CR1-expressing immune cells results in profound attenuation of tumor progression after radiation. Together, our results suggest that the immune system, using CX3CR1 signaling, plays a crucial role in radiation resistance in the LLC mouse model. 

CX3CR1 is expressed by many immune cells, including tissue-resident macrophages and dendritic cells, and infiltrating immune cells such as monocytes, CD8^+^ T cells, and NK cells [9]. Infiltration of CX3CR1 expressing NK cells, dendritic cells, CD4^+^ and CD8^+^ T cells into the tumor results in an increase in the antitumor immune response, reduces tumor growth, and increases the survival of mice and cancer patients [26,27,28,29]. However, CX3CR1 is also highly expressed on monocytes and macrophages in the TME and may play an opposing role to that described. TAM are the most abundant cells in the TME and display a spectrum of phenotypes broadly categorized into two subsets, namely, M1 antitumor and M2 pro-tumor phenotypes [30]. M1 macrophages express high antigen presentation and secret proinflammatory cytokines like IL-12, IL-23, TNF-α, and reactive oxygen species. On the other hand, M2 macrophages play a significant role in immune suppression, angiogenesis, and tumor progression by secreting various immunomodulatory soluble factors, including IL-6, IL-10, and TGF-β [30,31]. Previous studies showed that CX3CR1-expressing macrophages display an M2-like phenotype [32], promoting angiogenesis and liver metastasis in mouse colon cancer models and conferring poor prognosis in cancer patients [33]. In line with our results, Schmall et al. showed that TAM depletion and genetic ablation of CX3CR1 inhibited LLC growth and metastasis and shifted macrophages toward an anti-tumorigenic phenotype [34]. These results suggest that CX3CR1 plays both a pro and an anti-tumorigenic role in different immune cells, cancers, and other TME exposures. 

One of the significant hallmarks of cancer is uncontrolled proliferation and resistance to apoptosis [35]. Growing evidence implicates CX3CL1–CX3CR1 signaling in directly stimulating cancer cell proliferation, as has been shown in gastric cancer [13], pancreatic cancer [14], and breast cancer cells [36]. Our study further supports the CX3CL1–CX3CR1 axis role in cancer cell proliferation and apoptosis following radiation. It amplifies that this effect is also mediated by CX3CR1 signaling in the immune microenvironment and not solely by directly impacting cancer cells. Interestingly, Tang et al. [37] showed that CX3CL1 induced DU145 prostate cancer cell proliferation and promoted the G1–S-phase transition by upregulating the expression levels of cyclin E and CDK2. A possible explanation may be that depleting CX3CR1 expressing immune cells in the culture increases the CX3CL1 available for cancer cells, promoting the G1–S-phase transition.

The reduced proliferation and increased apoptosis observed in irradiated LLC cells co-cultured with CX3CR1-deficient macrophages could be partly due to the dysregulation of cytokines, including the reduction in IL-6 secretion and the increase in IL-1α and MCP-1. It is well-established that IL-6 promotes cancer cell proliferation through its receptor on the surface of cancer cells by activating intracellular signaling pathways that support cell growth [38]. IL-6 also regulates apoptosis by activating STAT-3 and NF-kB signaling, which transactivates the expression of many anti-apoptotic proteins, including Bcl-2, Bcl-xL, and Mcl-1 [38,39,40,41]. In addition, our results are supported by studies on various cancer cell lines, such as MCF-7 breast cancer, A375 melanoma, prostate stem cells, and murine primary mammary cells, which found that IL-1α inhibits cancer cell proliferation and induces G0–G1 cell cycle arrest [42,43,44]. Interestingly, we observed an upregulation in the macrophage chemoattractant protein MCP-1 in CX3CR1-deficient macrophages. Other studies have shown that mononuclear cells isolated from the eyes of CX3CR1-deficient mice have a robust upregulation of MCP-1 expression [45]. Importantly, MCP-1 is a key regulator of CX3CR1 expression in macrophages and enhances its expression and adhesion to CX3CL1 [46]. Thus, our findings suggest that blocking CX3CR1 may lead to a compensatory mechanism to attract mononuclear cells via MCP-1 signaling.

Recently, CX3CR1-blocking small molecules [47] and anti-CX3CL1 monoclonal antibodies [48,49,50] have been developed and are currently being tested in clinical trials to modulate the inflammatory process associated with myocardial infarction, rheumatic arthritis [48,49,50], and COVID-19 infection. To date, blocking the CX3CL1–CX3CR1 axis has not been tested in human cancer patients. However, a recent study has shown that treating ovarian cancer cells with the small molecule CX3CR1 inhibitor, KAND567 results in sensitization to platinum drugs, replication fork stalling, impaired cell cycle progression, and unresolved cisplatin–DNA adducts [51]. Our study complements these results and shows that CX3CR1 inhibition may also increase the tumor’s sensitivity to radiation treatment by modulating the immune microenvironment. Therefore, a potential implication of our study is that it creates the scientific rationale for pairing CX3CR1 inhibitors with radiation as a novel therapeutic strategy for treating lung cancer patients.

We are aware of several limitations in our work. First, the number of irradiated LLC colonies did not reach a statistically significant difference when the cancer cells were co-cultured with CX3CR1-deficient macrophages compared to WT. One possible explanation is that the concentration of CX3CL1 may have been sufficient to activate the WT macrophages and trigger the changes in RNA expression of cell cycle-associated genes; however, it is not adequate to translate to differences in the proliferation of the irradiated LLC cells. In addition, while there was a significant difference in the secretion of IL-1α, MCP-1, and IL-6 between WT and CX3CR1-deficient macrophages in vitro, adding FKN to the medium did not affect the secretion in WT macrophages. A possible explanation is that the concentration of CX3CL1 in the medium of the WT macrophages was already at saturation point due to macrophages’ CX3CL1 secretion. Therefore, no additional benefit was obtained from the administration of the exogenous ligand. Further studies are required to understand the receptor–ligand kinetics of CX3CR1–CX3CL1. Our study also investigated CX3CR1 expressing macrophages associated with tumors using peritoneal macrophages from CX3CR1-deficient and WT mice. Peritoneal macrophages are a common tool utilized to study macrophage function in various models and in vitro assays due to their being mature, easy to obtain with minimum tissue manipulation, and with high expression of inducible cytokines [52,53]. However, it is important to acknowledge that there may be inherent differences between peritoneal macrophages and TAMs in functionality and response to environmental signals. These differences should be considered when interpreting the results of our experiments. In addition, our study focused on LLC, a commonly used mouse adenocarcinoma lung cancer model. Therefore, our results may only apply to this model and need to be validated in other cancer models.

## 5. Conclusions

In summary, CX3CR1-expressing immune cells invade the TME after radiation therapy in a mouse lung cancer model. CX3CR1 cell depletion attenuates tumor growth following radiation and sensitizes the tumor to S-phase-specific chemotherapy. A deeper understanding of the role of CX3CR1 in the irradiated TME may ultimately pave the way for the development of a novel therapy combining CX3CR1 targeted immunotherapy and radiation. 

## Data Availability

The datasets used and/or analyzed during the current study are available from the corresponding author on reasonable request.

## Supplementary Materials

The following supporting information can be downloaded at: https://www.mdpi.com/article/10.3390/cancers15225472/s1, Figures S1–S4; Table S1.
