# Supplementary material for: CX3CR1-Expressing Immune Cells Infiltrate the Tumor Microenvironment and Promote Radiation Resistance in a Mouse Model of Lung Cancer
Source: Cancers (Basel). 2023 Nov 19;15(22):5472. doi: 10.3390/cancers15225472 (PMC10669975; doi:10.3390/cancers15225472)
Supplement: Supplementary file 1 [file cancers-15-05472-s001.zip › Table S1.pdf]

Supplementary Table S1: List of primers:

CDK1 forward		AGGTACTTACGGTGTGGTGTAT
CDK1 reverse		CTCGCTTTCAAGTCTGATCTTCT
CDK2 forward		CTCGACACTGAGACTGAAGGT
CDK2 reverse		GCAGCTTGACGATATTAGGGTGA
CDK4 forward		AAGGTCACCCTAGTGTTTGAGC
CDK4 reverse		CCGCTTAGAAACTGACGCATTAG
Cyclin A2 forward		GCCTTCACCATTCATGTGGAT
Cyclin A2 reverse		TTGCTCCGGGTAAAGAGACAG
Cyclin B1 forward		GCGTGTGCCTGTGACAGTTA
Cyclin B1 reverse		CCTAGCGTTTTTGCTTCCCTT
Cyclin D1 forward		GCGTACCCTGACACCAATCTC
Cyclin D1 reverse		ACTTGAAGTAAGATACGGAGGGC
Cyclin E1 forward		GTGGCTCCGACCTTTCAGTC
Cyclin E1 reverse		CACAGTCTTGTCAATCTTGGCA
GAPDH forward		TTGAGGTCAATGAAGGGGTC
GAPDH reverse		TCGTCCCGTAGACAAAATGG
