# Supplementary figures and images for: CX3CR1-Expressing Immune Cells Infiltrate the Tumor Microenvironment and Promote Radiation Resistance in a Mouse Model of Lung Cancer
Source: Cancers (Basel). 2023 Nov 19;15(22):5472. doi: 10.3390/cancers15225472 (PMC10669975; doi:10.3390/cancers15225472)

Supplementary Figure S1

A.

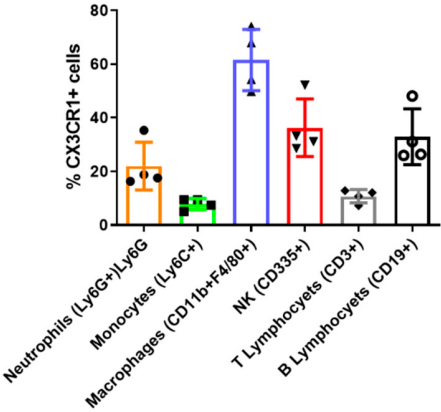

B.

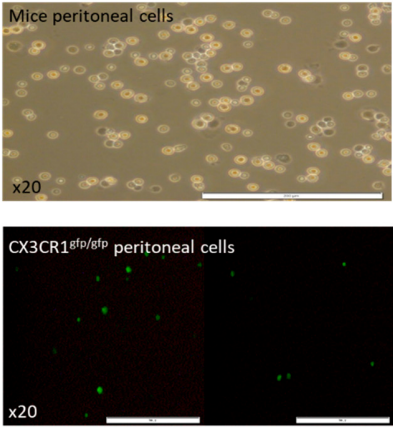

Supplement: Supplementary file 1 [file cancers-15-05472-s001.zip › Figure S1.pdf]

Supplementary Figure S2

A.

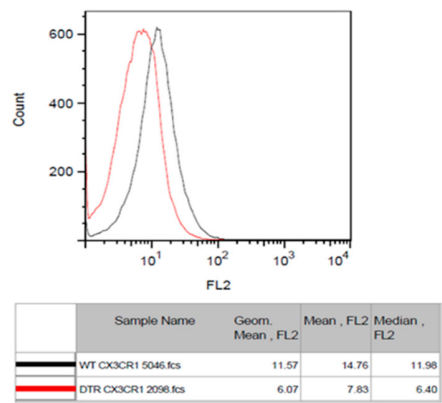

B.

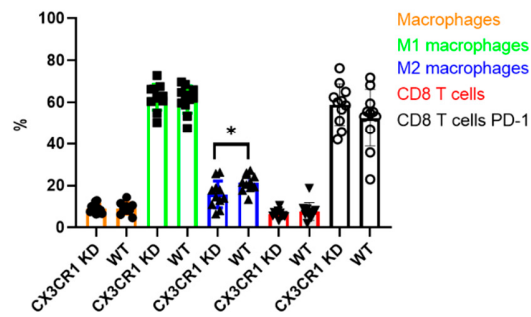

Supplement: Supplementary file 1 [file cancers-15-05472-s001.zip › Figure S2.pdf]

Supplementary Figure S3

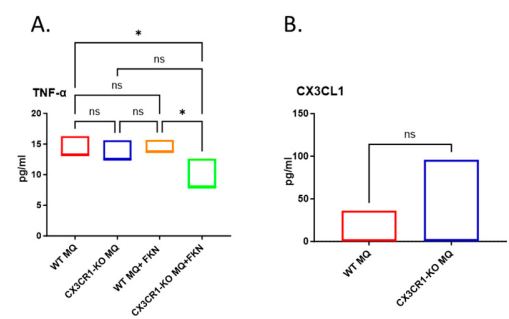

Supplement: Supplementary file 1 [file cancers-15-05472-s001.zip › Figure S3.pdf]

## Supplementary Figure S4

A.

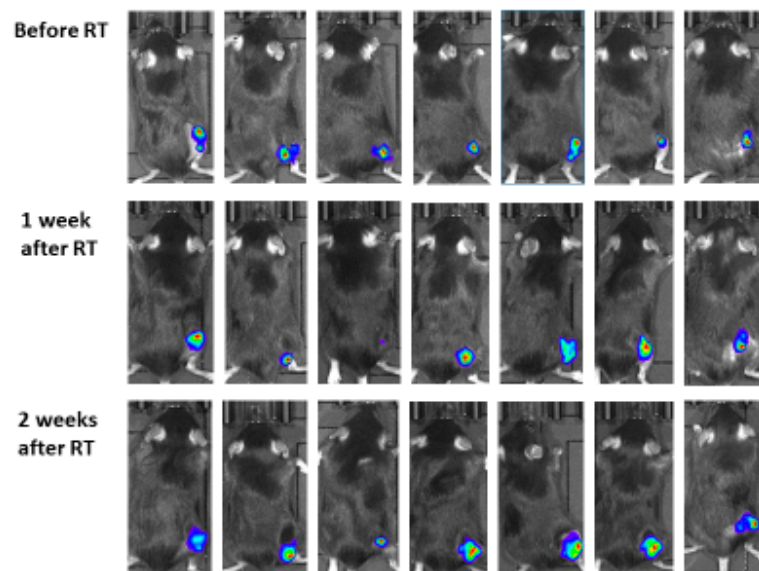

B.

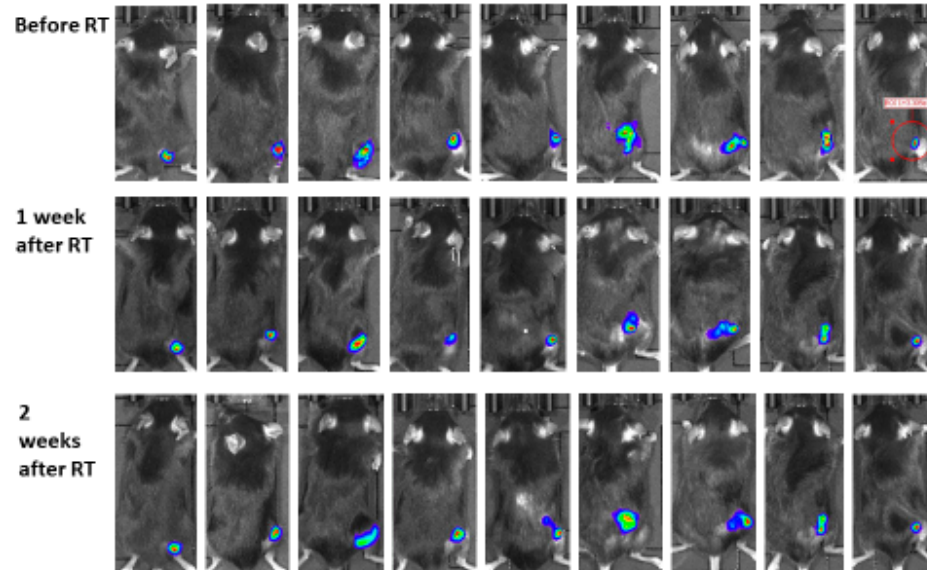

C.

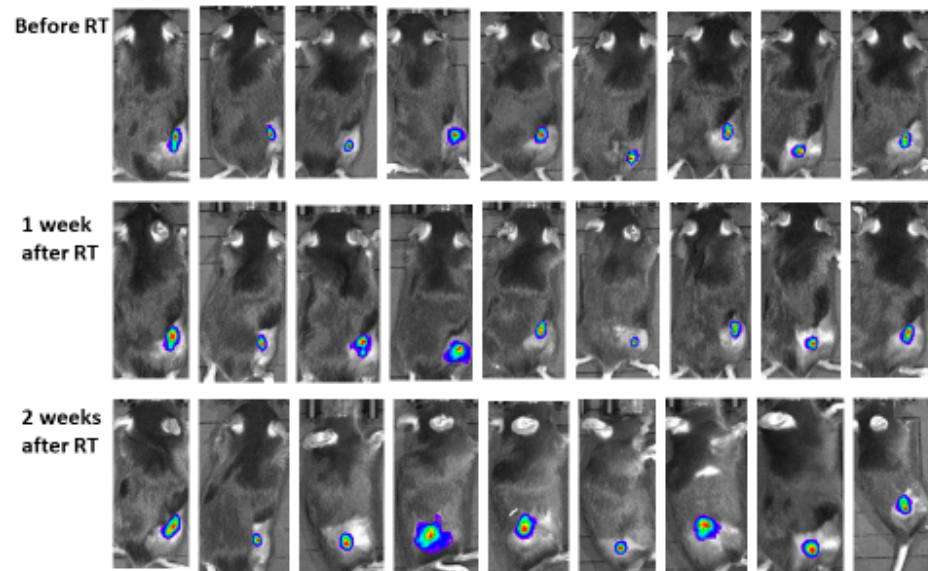

D.

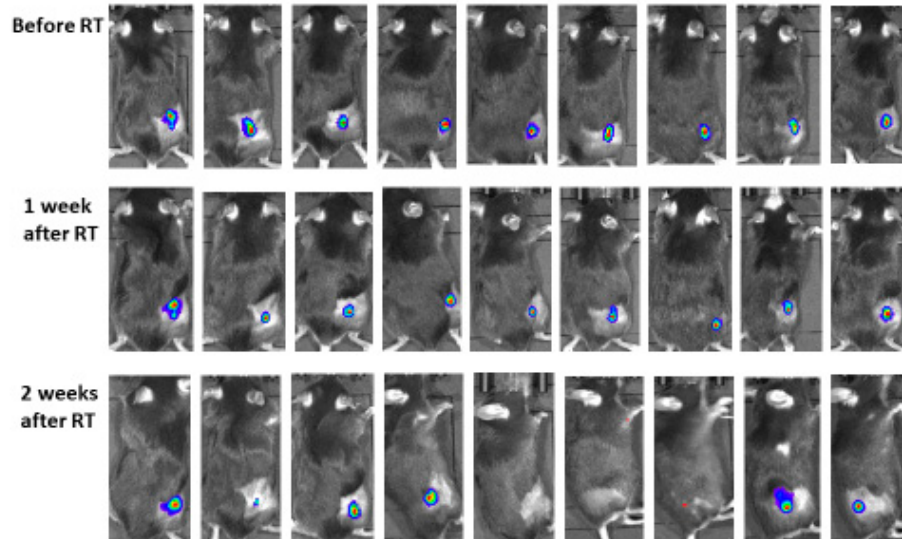

Supplement: Supplementary file 1 [file cancers-15-05472-s001.zip › Figure S4.pdf]
